# Draft Genome Sequence of *Campylobacter jejuni* CAM970 and *C. coli* CAM962, Associated with a Large Outbreak of Foodborne Illness in Fukuoka, Japan, in 2016

**DOI:** 10.1128/genomeA.00508-17

**Published:** 2017-06-15

**Authors:** Hiroshi Asakura, Naoto Takahashi, Shiori Yamamoto, Hiroyuki Maruyama

**Affiliations:** a Division of Biomedical Food Research, National Institute of Health Sciences, Tokyo, Japan; b Health Science Section, Fukuoka City Institute of Health and Environment, Fukuoka, Japan

## Abstract

Here, we report the draft genome sequences of *Campylobacter jejuni* CAM970 and *C. coli* CAM962, which were associated with a large outbreak of foodborne illness originating from undercooked chicken sushi in Fukuoka, Japan, in May 2016. Their genome sizes were 1,690,901 and 1,704,736 bp, with 22 and 23 rRNAs, 9 and 9 tRNAs, and 411× and 419× coverage for *C. jejuni* CAM970 and *C. coli* CAM962, respectively.

## GENOME ANNOUNCEMENT

*Campylobacter jejuni* and *C. coli* are two of the leading causes of foodborne gastrointestinal illnesses, which, worldwide, number approximately 400 to 500 million cases annually (1, 2). In Japan, a total of 339 cases of foodborne campylobacteriosis were reported in 2016, which represented 29.7% of all foodborne cases (1,140 cases) reported that year (3). Epidemiological data have shown that chicken meat is one of the main sources for human campylobacteriosis (4). In May 2016, a large foodborne outbreak caused by *Campylobacter* spp. occurred; 266 patients suffered from gastroenteritis due to the consumption of undercooked chicken meat and vinegar rice (sushi) in Fukuoka city. Bacteriological surveillance determined that both *C. jejuni* and *C. coli* were associated with the outbreak. In order to characterize their genomic traits, we examined, and report here, the draft genome sequences of *C. jejuni* CAM970 and *C. coli* CAM962, which were isolated from patients treated during the outbreak. Genomic DNA of the two strains was sequenced by single-end sequencing with an Ion Torrent PGM sequencer (Thermo Fisher Scientific, Waltham, MA, USA), resulting in average coverages of 411× and 419×, respectively. Raw reads were trimmed and *de novo* assembled using CLC Genomics Workbench version 9.0 (Qiagen, Hilden, Germany). The parameters for trimming were as follows: ambiguous limit, 2; quality limit, 0.05; number of 5′ terminal nucleotides, 20; and number of 3′ terminal nucleotides, 5. The parameters for the *de novo* assembly were as follows: mapping mode, create simple contig sequences (fast); bubble size, 50; word size, 21; minimum contig length, 500 bp; perform scaffolding, no; and autodetect paired distances, yes. The draft genomes of *C. jejuni* CAM970 and *C. coli* CAM962 were assembled into 61 and 34 contigs with accumulated lengths of 1,690,901 and 1,704,736 bp (*N*_50_, 92,774 and 156,200 bp) and average G+C contents of 30.2 or 31.3%, respectively. The genome was annotated with the RAST server (5). Annotation of these assemblies identified 1,813 and 1,820 coding sequences, 22 and 23 rRNAs, and 9 and 9 tRNAs, for *C. jejuni* CAM970 and *C. coli* CAM962, respectively.

The sequence types (STs) of these strains were also profiled with a multilocus sequence typing (MLST) scheme (6). The results showed that strains CAM970 and CAM962 belonged to ST5716 and ST1181, respectively, and both strains were found to associate with human infection based on the *Campylobacter* MLST database (https://pubmlst.org/campylobacter). The data provided here can aid in future efforts to identify the sources of infections, since the genome sequence-based framework has potential advantages for epidemiological studies of *Campylobacter* infections (7). With the increased application of whole-genome sequencing technologies to epidemiological studies of *Campylobacter* outbreaks (i.e., source tracking) (8, 9), further accumulation of genomic data of *Campylobacter* spp. associated with foodborne outbreaks will contribute to improving our understanding of host and geographic adaptation of this pathogen and lead to the development of preventive strategies.

### Accession number(s).

This whole-genome shotgun project has been deposited at DDBJ/EMBL/GenBank under the accession numbers BDRZ01000000 (CAM970) and BDRY01000000 (CAM962). The versions described in this paper are the first versions, BDRZ01000000 (CAM970) and BDRY01000000 (CAM962).
